# Thermal and Modern, Non-Thermal Method Induction as a Factor of Modification of Inulin Hydrogel Properties

**DOI:** 10.3390/foods12224154

**Published:** 2023-11-17

**Authors:** Anna Florowska, Tomasz Florowski, Bartosz Kruszewski, Emilia Janiszewska-Turak, Weronika Bykowska, Nour Ksibi

**Affiliations:** 1 Department of Food Technology and Assessment, Institute of Food Sciences, Warsaw University of Life Sciences-SGGW, 159c Nowoursynowska Street, 02-787 Warsaw, Poland; tomasz_florowski@sggw.edu.pl (T.F.); bartosz_kruszewski@sggw.edu.pl (B.K.); w.bykowska98@gmail.com (W.B.); 2 Department of Food Engineering and Process Management, Institute of Food Sciences, Warsaw University of Life Sciences-SGGW, 159c Nowoursynowska Street, 02-787 Warsaw, Poland; emilia_janiszewska-turak@sggw.edu.pl; 3 Faculty of Sciences of Tunis, Tunis El Manar University, El Manar Tunis 2092, Tunisia; 4 Laboratory of Aromatic and Medicinal Plants (LPAM), Centre of Biotechnology of Borj Cedria, BP. 901, Hammam-Lif 2050, Tunisia

**Keywords:** inulin hydrogel, ultrasounds, HPH, HHP

## Abstract

The aim of the study was to compare the properties of inulin hydrogels obtained with different methods, e.g., the traditional–thermal method and new, non-thermal methods, used in food production, like ultrasonic, high-pressure homogenization (HPH), and high hydrostatic pressures (HHPs). It was found that each of the compared induction methods allowed for obtaining inulin hydrogels. However, the use of non-thermal induction methods allows for obtaining a gel structure faster than in the case of thermal induction. In addition, hydrogels obtained with new, non-thermal methods differ from gels obtained with thermal treatment. They were characterized by higher stability (from 1.7 percent point-of-stability parameters for HHP 150 MPa to 18.8 for HPH II cycles) and in most cases, by improved microrheological properties–lower solid–liquid balance toward the solid phase, increased elasticity and viscosity indexes, and lowering the flow index. The gels obtained with the new, non-thermal method were also characterized by a more delicate structure, including lower firmness (the differences between thermal and non-thermal inductions were from 0.73 N for HHP at 500 MPa to 2.39 N for HHP at 150 MPa) and spreadability (the differences between thermal and non-thermal inductions were from 7.60 Ns for HHP at 500 MPa to 15.08 Ns for HHP at 150 MPa). The color of ultrasound-induced inulin gels, regarding the HPH and HHP technique, was darker (the differences in the L* parameter between thermal and non-thermal inductions were from 1.92 for HHP at 500 MPa to 4.37 for 10 min ultrasounds) and with a lower a* color parameter (the differences in the a* parameter between thermal and non-thermal inductions were from 0.16 for HHP at 500 MPa to 0.39 for HPH II cycles) and b* color parameter (the differences in the b* parameter between thermal and non-thermal inductions were from 1.69 for 5 min ultrasounds to 2.68 for HPH II cycles). It was also found that among the compared induction methods, the high-pressure technique has the greatest potential for modifying the properties of the created inulin hydrogels. Thanks to its application, depending on the amount of applied pressure, it was possible to obtain gels with very different characteristics, both delicate (i.e., soft and spreadable), using HHP at 150 MPa, and hard, using HHP at 500 MPa, the closest in characteristics to gels induced with the thermal method. This may allow the properties of hydrogels to be matched to the characteristics of the food matrix being created.

## 1. Introduction

Inulin is a linear polydisperse carbohydrate, composed of monomers of fructose linked mainly by β-(2–1) glycosidic linkages [1], that has an ability to form a hydrogel structure [2]. For the production of hydrogel structures, the induction of this process is essential. One of the most popular and simple induction methods is thermal induction. It belongs to the physical cross-linking methods and involves heating and then cooling the water solution. The primary mechanism for the formation of hydrogels by the action of an elevated temperature is the transition of the sol to gel. In order for the gelling process to begin, the aqueous solution containing the polymers must be heated to a temperature above the dissolution point [3,4,5]. When the solution is heated, the sol structure becomes disordered and the solution begins to swell. The next step in this method, which is cooling the solutions, leads to an ordered polymer network. The thermal induction of gelation leads to the formation of physical interactions between the polymer molecules, such as hydrogen bonds and ionic bonds [3,4,6]. The most popular parameters for inulin gelation for thermal induction are a temperature of 80 °C and an inulin concentration of 20 g/100 g [7,8]. Although the thermal method is a simple and widely known method of inducing hydrogels, it has its limitations. The thermal method cannot be used in products where no heating is applied. In such situations, food producers have to resort to other, non-thermal methods.

Such new, non-thermal food processing methods, increasingly used in food production, which are indicated in the literature to allow the induction of inulin gelation, include ultrasonic treatment [9], high-pressure homogenization [10], and the use of high hydrostatic pressures [11].

The use of ultrasound in the formation of hydrogels is a new type of physical modification to obtain gels. It is a safe and environmentally friendly method that allows for obtaining gels with a more homogeneous and denser network structure, which affects their stability [12,13]. The positive effect of ultrasound on the induction of gelation is visible in the improvement of the solubility of polymer solutions, and in cases where the bases of hydrogels are compounds with insoluble components, ultrasonic treatment causes almost complete disappearance of insoluble fractions, which greatly facilitates the production of gels. Ultrasound also affects the degree of swelling; it is higher compared to induction with the thermal method. As a result of ultrasonic polymerization, hydrogels are characterized by a more averaged and reproducible particle size. This method can also function to support gelation used in other methods [14,15]. Ultrasonic treatment promotes the formation of hydrogen bonds between the molecules [9].

The induction of inulin hydrogels can also be performed by applying shear forces on solutions and during the homogenization, the hydrogen bonds and van der Waals interactions among particles in the dispersion (aggregates of molecules) are formed [8,16]. One of the methods, where such shear forces are used, is high-pressure homogenization (HPH), in which the solution in a continuous flow is subjected to high pressures in the range of 50 MPa to 400 MPa [17]. During HPH, the liquid is forced through the small gap of the valve by high pressure, which creates a large mechanical force on the liquid [18]. The gel structure is induced by physical forces, including cavitation, turbulence, impingement, shear stress, pressure gradient, as well as extensional shear, responsible for the reinforced structures [19]. High-pressure homogenization is a process that increases the solubility and water-holding capacity of HPH-treated solutions [20] and causes structural modifications of polysaccharides [21]. These properties are of great importance in the gelation process, which is why induction with high-pressure homogenization is a new, very promising technology for the formation of hydrogels [21].

Higher pressures than in the HPH method have also been attempted to induce gelation of polysaccharides. High hydrostatic pressure (HHP) treatment is a non-thermal process carried out at a pressure up to 1000 MPa, which is widely used (typically at pressures of 500–600 MPa) to inactivate pathogenic microorganisms without causing large losses of nutrients [22]. HHP can break or change non-covalent interactions (e.g., hydrogen bonds and van der Waals forces) in polymer molecules with minimal impact on covalent bonds. Inducing changes in food polymers leads to the start of the gelation process [23]. High hydrostatic pressures limit the movement of molecules, and when the hydrogen bonds are broken, the molecules become packed to fill the spaces between them, which leads to harder hydrogels compared to those induced by heat [24]. In addition, it has been shown that HHP, compared to conventional induction methods, has a positive effect on the strength and viscoelasticity of gels and their water-holding capacity [23].

The available literature lacks a comprehensive comparison of the properties of hydrogels obtained with different methods; therefore, the aim of the article is to compare the properties of inulin hydrogels obtained with non-thermal methods: ultrasonic, high-pressure homogenization, and high hydrostatic pressures with the thermal one. This research can therefore provide valuable information for food producers, including functional food producers, on the possibility of modifying the properties of final products based on a polysaccharide hydrogel matrix.

## 2. Materials and Methods

The research material was inulin (HPX, Beneo GmbH, Mannheim, Germany) used in a concentration of 20 g/100 g [16] to form hydrogel induced with the thermal method (inulin was dissolved in water at 80 °C and cooled), ultrasounds (inulin dispersed in water at 20 ± 1 °C was sonicated for 5 and 10 min (25 kHz, 70 W, 100% pulse, 100% amplitude, sonotrode immersion at 15 mm) using a UP200St ultrasonic homogenizer (Hielscher Ultrasonics GmbH, Teltow, Germany) equipped with a titanium sonotrode S26d7), high-pressure homogenization (HPH) (inulin dispersed in water at 20 ± 1 °C was subjected to high-pressure homogenization at a pressure of 100 MPa in I or II cycles in the GEA Lab Homogenizer Twin PANDA 600 (Parma, Italy)), and high hydrostatic pressure (HHP) treatment (inulin dispersed in water at 20 ± 1 °C was subjected to high hydrostatic pressure for 10 min at a pressure of 150 MPa and 500 MPa; the process was carried out in the Pascalizer U5000/120 Unipress, Warsaw, Poland). All the induction method parameters were chosen according to the evadible literature data for the specific condition required to form inulin gels [7,11,12,25]. After induction, the hydrogels were stored under refrigeration conditions (8 ± 1 °C) for 24 h in order to obtain a stable structure. After this time, the samples were conditioned to a temperature of 20 ± 1 °C; then, the properties of hydrogels were tested, with the exception of the measurement of microrheological properties, which was carried out immediately after induction.

The methods used to compare the properties of hydrogels obtained with various induction methods were the following:

Volumetric Gel Index (VGI): expresses the degree of gel formation as the volume of gel over the total volume of the sample. The sol, after induction with the tested method, was poured into a cylinder and left for 24 h at a temperature of 20± 1 °C. After this time, the VGI was calculated. VGI equaling 0 means that no gel was formed; VGI between 0 and 99% pictures partial gel formation, and VGI equaling 100% means that the gel structure is completely formed [16].

Physical Stability: the test analyzed the physical stability of the sample based on the amount of leakage obtained during the centrifugation of the sample. Equipment: centrifuge MPW-352 (MPW, Warsaw, Poland), parameters: volume of the dispersion—10 mL; 5000 rpm; an experiment time of 15 min; a temperature of 20 ± 1 °C. Physical stability was expressed as the volume of gel after leakage removal relative to the total volume of the sample.

Microstructure: in order to test the microstructure of hydrogels, the freeze-dried samples were placed on double sticky tape, coated with a thin layer of gold, and placed in an electron scanning microscope (Hitachi TM3000, Hitachi, Japan). The microstructure was analyzed under an accelerating voltage of 15 kV, at pressures of 100 Pa and a magnification of ×3000.

Textural Properties: measured at 20 ± 1 °C using texture analyzer TA.XT Plus (Stable Micro Mixtures, Surrey, UK), equipped with a 0.5-cm-diameter cylindrical flat probe (P/0.5R) to measure the hydrogels’ firmness (N) and adhesiveness (N·s) (parameters: test speed—1.0 mm/s, penetration depth—8 mm), and equipped with the TTC Spreadability Rig to measure the hydrogels’ spreadability (N·s), (parameters: test speed—3.0 mm/s).

Microrheological Properties: the microrheological properties of the hydrogels were investigated with the multi-speckle diffusing-wave spectroscopy method (MS-DWS), using a Rheolaser Master device (Formulaction, Toulouse, France), and the following parameters: samples placed in glass vials—20 mL, wavelength—650 nm, time—23 h, temperature—20 ± 1 °C. The detector captures the interfering backscattered waves, and the measurement results were recorded using Rheotest software RheoSoft Master_v1.4.0.10 [26]. The parameters that were determined are Mean Square Displacement (MSD) (nm^2^) curves, Elasticity Index (EI) (nm^−2^), solid–liquid balance (SLB), Macroscopic Viscosity Index (MVI) (nm^−2^·s), and Fluidity Index (Hz).

Color Parameters: equipment—Minolta CR-400 colorimeter (Minolta, Tokyo, Japan; light source D65, and a measuring head hole of 8 mm). The color components were measured in the CIEL*a*b* system. Based on the color measurement, three color components—L* (brightness), a*, and b* color components—were calculated. With these three color components, the color difference coefficient can be calculated and expressed as delta E (ΔE). This factor determines how much two colors differ from each other, and is calculated from the following formula [27]:

$$\Delta E=\sqrt{{\left(L_{1}^{*}-L_{2}^{*}\right)}^{2}+{\left(a_{1}^{*}-a_{2}^{*}\right)}^{2}+{\left(b_{1}^{*}-b_{2}^{*}\right)}^{2}}$$

where:

Δ*E*—color difference coefficient,

$L_{1}^{*},\;a_{1}^{*},b_{1}^{*}$ and $L_{2}^{*},\;a_{2}^{*},b_{2}^{*}$ refer to the color parameters of compared hydrogels.

Depending on the Δ*E* values, the color difference between the samples can be estimated as not noticeable for the observer (when 0 < ∆*E* < 1), only an experienced observer can notice the difference (1 < ∆*E* < 2), an unexperienced observer also notices the difference (2 < ∆*E* < 3.5), a clear difference in color is noticed (3.5 < ∆*E* < 5), and the observer notices two different colors (5 < ∆*E*) [27].

Statistical Analysis: the gathered data from three independent, experimental repetitions (*n* = 3) were statistically evaluated using Statistica 13.3 (TIBCO Software Inc., Palo Alto, CA, USA) software. In order to assess the significance of differences in the average values of measured parameters of hydrogels, a one-way analysis of variance was performed. Tukey’s test at a significant level of α = 0.05 revealed significant differences between hydrogels obtained in different induction methods.

## 3. Results and Discussion

### 3.1. The Influence of Induction Methods on Formation and Stability of Inulin Hydrogels

**Figure 1:**
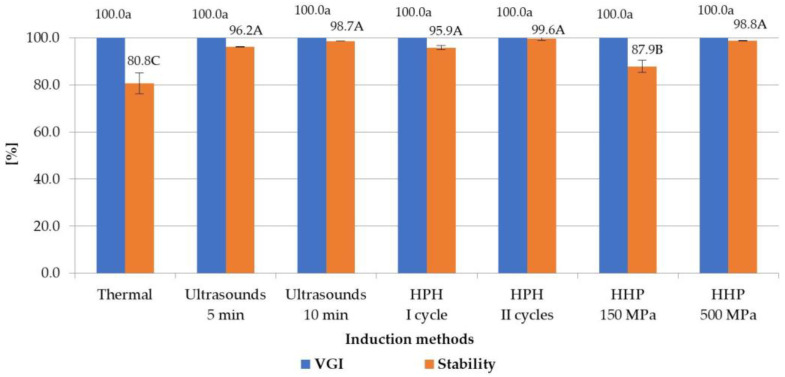
Effect of induction method on VGI and stability of inulin hydrogels (the average (*n* = 3) values marked with different letter symbols differ significantly (*p* < 0.05)).

In order to investigate the influence of selected induction methods on the properties of hydrogels, the degree of gel structure formation (VGI) was assessed. VGI values are presented in Figure 1. All tested induction methods allowed for obtaining a VGI of 100%. This means that each of the prepared solutions was completely gelled within 24 h at 8 °C, regardless of the induction method used. However, the mechanism of creating the gel network for each method was different. In the case of the thermal induction, those responsible for the gelation effect were the network structures that were formed, after dissolution of inulin, among the molecular chains through entanglement of molecules [28], whereas during the ultrasound treatment for polysaccharides the gelation effect is probably due to the cavitation effect of ultrasounds [29]. The high-pressure homogenization results in increasement of the solubilization and dispersion of the inulin powder, producing small crystals with enhanced interactions with other inulin particles and water molecules [10]. As for high hydrostatic pressure, the gel induction process is similar to the mechanically induced gels—by the intermolecular hydrogen bonds, which are probably mediated through the bridging water molecules in cross-linking junctions [11].

The stability of the structures of the obtained gels is also, along with the ability to create a gel structure, an extremely important indicator of the quality and application of a given hydrogel. It was found that the applying of new, non-thermal processing methods for the induction of hydrogels allowed for obtaining inulin hydrogels with significantly higher stability than gels obtained using the traditional, thermal method (from 1.7 percent point-of-stability parameters for HHP at 150 MPa to 18.8 for HPH II cycles) (Figure 1). At the same time, it was found that the compared non-thermal induction methods allowed, in most cases, for obtaining gels with similar stability. Only gels induced by high hydrostatic pressure, with lower parameters (i.e., 150 MPa), were characterized by slightly lower stability than other not-thermally indicated gels (differences between HHP and other not-thermal methods ranged from 8.0 percent point-of-stability parameters for HPH I cycle to 11.7 for HPH II cycles). In the case of gelation induction with high hydrostatic pressure, an increase in the stability of hydrogels was observed with the increase in the pressure used for their processing. At the induction pressure of 500 MPa, the stability of gels was 98.8%, which was more than 10 percent points higher than the stability of the gels obtained at the pressure of 150 MPa. It was also observed that in the case of ultrasonic and HPH treatment, the changing process parameters had no significant effect on the stability of the obtained gels.

The positive effect of ultrasonic induction on the stability of hydrogels was also found by Zang et al. [13] and Mekala et al. [30], who studied the mechanisms of the induction of pectin gels. According to the authors, the higher stability of ultrasound-induced hydrogels compared to thermally induced gels may be due to the increased solubility of polymers and the formation of a more homogeneous and denser network structure. Higher stability of hydrogels after high-pressure homogenization of inulin solvents was also demonstrated by Alvarez-Sabatel et al. [10]; according to the authors, this can be explained by the formation of intermolecular interactions, which build a more stable network structure. An increase in stability when using high hydrostatic pressures in relation to thermal induction of hydrogels was shown by Florowska et al. [11] for gels based on inulin, Li and Zhu [31] for starch, and Joile et al. [32] for pectins. In turn, the increase in the stability of hydrogels with the increase in hydrostatic pressure from 150 to 300 MPa was shown also by Florowska et al. [24], who studied the effect of high-pressure induction parameters on the properties of inulin hydrogels.

### 3.2. The Influence of Induction Methods on Microstructure and Texture of Inulin Hydrogels

**Figure 2:**
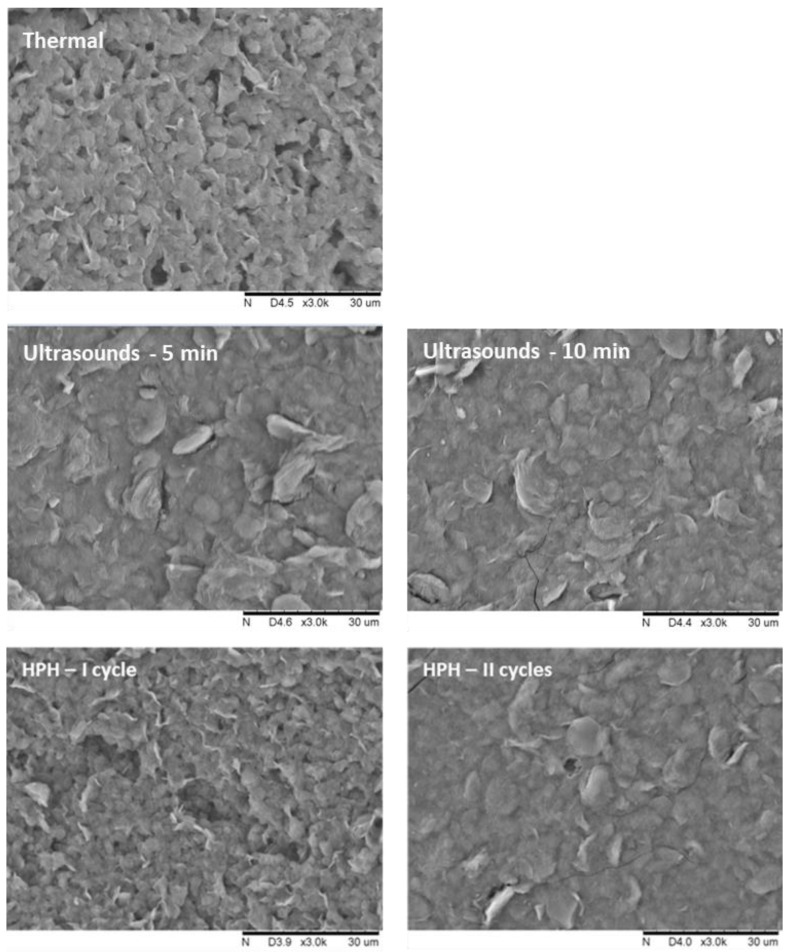

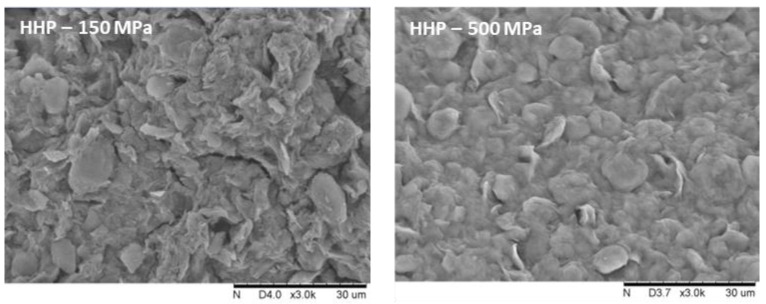
Effect of induction method on the microstructure of inulin hydrogels (magnification ×3000).

Based on the analysis of SEM microscopic images, it can be concluded that the gels obtained with non-thermal methods have a more flattened structure, and the visible particles are larger, but the structure is more compact (Figure 2). It was also noticed that the applied induction parameters for individual methods also had an impact on the structure of the hydrogels. First of all, it was observed that for a longer time of high-pressure treatment, two HPH cycles or higher HHP pressure, the samples had a more uniform and smoother structure, which is characterized also by lower porosity. These differences in the microstructure of inulin hydrogels induced with different methods were reflected in the measured texture parameters.

**Figure 3:**
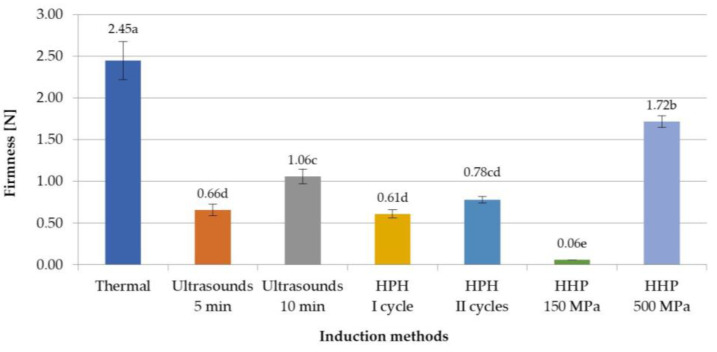
Effect of induction method on firmness of inulin hydrogels (the average values (*n* = 3) marked with different letter symbols differ significantly (*p* < 0.05)).

The texture analysis of inulin hydrogels included firmness, adhesiveness, and spreadability. It was stated that the firmness of inulin hydrogels induced with non-thermal methods was much lower than that of gels obtained with thermal methods (Figure 3). This indicates that obtaining a hydrogel induced with non-thermal methods with a firmness similar to that of thermally induced gels will require strengthening their structure. According to research by Florowska et al. [33,34], such strengthening of the structure of inulin hydrogels can be achieved, among others, by the addition of proteins or other polysaccharides. Analyzing the influence of induction parameters on firmness, it was also found that the differences between the firmness parameter between thermal and non-thermal induction methods were from 0.73 N for HHP at 500 MPa to 2.39 N for HHP at 150 MPa. It was also found that in the case of ultrasound and HHP induction, variable process parameters, i.e., time of ultrasound treatment or level of pressure, had a significant impact on this texture parameter. In the case of ultrasound induction, the differences in time treatment between 5 and 10 min were 0.40 N, whereas gels subjected to a pressure of 500 MPa were characterized by a significantly higher value, by 1.66 N, of this parameter compared to induction with a pressure of 150 MPa. This is the result of a greater reduction in the movement of molecules and breaking more hydrogen bonds, so that the molecules are packed to fill the voids, resulting in an increase in hardness, less observed at lower pressure (150 MPa) [24]. This means that the HHP pressure of 150 MPa is sufficient to form a gel structure, but does not allow for a creation of a strong network between the polymers. In the case of HPH induction methods, there was no significant impact of different induction process parameters on the firmness of inulin hydrogels.

**Figure 4:**
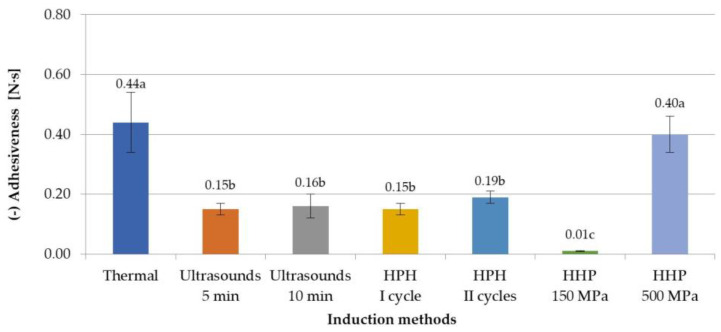
Effect of induction method on adhesiveness of inulin hydrogels (the average values (*n* = 3) marked with different letter symbols differ significantly (*p* < 0.05)).

Examining the effect of selected induction methods on the adhesiveness of the obtained inulin gels, it was found that generally (except for gels induced with HHP at 500 MPa) a lower adhesiveness was obtained with non-thermal methods of hydrogel preparation (Figure 4). The differences between the adhesiveness parameter between thermal and non-thermal induction methods were from 0.25 Ns for HPH II cycles to 0.43 Ns for HHP at 150 MPa. The observations made by Lou et al. [9], in which authors were examining the influence of ultrasound on gelation ability of inulin, confirm the obtained results. It was further stated that the effect of the parameters of ultrasonic induction and high-pressure homogenization was not found. In the case of HHP-induced gels, the level of pressure used had an impact on the adhesives. The gels obtained using a pressure of 150 MPa were characterized by the lowest adhesiveness (−0.01 N·s), while those obtained using a pressure of 500 MPa had the highest adhesiveness (−0.40 N·s), similar to thermally induced gels. Florowska et al. [24] made similar observations of the effect of high pressure on the adhesiveness of inulin gels, comparing the influence of different HHP levels (150 and 300 MPa) and time treatment (5, 10, 20 min) on the gelation and properties of hydrogels with a different inulin concentration (15, 20, 25 g/100 g). The obtained results therefore indicate that very different gels can be obtained by using HHP by changing the process parameters.

**Figure 5:**
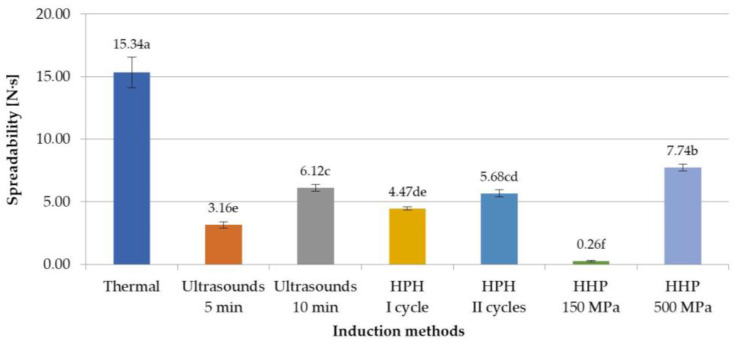
Effect of induction method on spreadability of inulin hydrogels (the average values (*n* = 3) marked with different letter symbols differ significantly (*p* < 0.05)).

Inulin gels, in the literature, are very often used as fat substitutes [35,36,37], which is why the effect of the induction method on the spreadability of obtained hydrogels was investigated. As with the other texture attributes that were evaluated in this study, it was found that spreadability depends on the induction method used (Figure 5). Lower values of this parameter were achieved with non-thermal-induced gels, which means that in practice they will be easier to spread. The spreadability of non-thermal samples was at least a half lower than hydrogels prepared with the thermal method. However, with the increase in parameters, i.e., the amount of applied pressure or the time of ultrasonic exposure, the value of the spreadability parameter increased (in case of HHP, the increase was of 7.48 N·s, and for ultrasounds, of 2.96 N·s). This can be explained by the fact that ultrasounds affected inulin solutions, improving the solubility of polymers, so extending the induction time from 5 min to 10 min increased the solubility of inulin, which resulted in the formation of a more ordered and thus stronger polymer network, more difficult to spread [13,30]. In the case of HHP, thanks to the use of different pressures (150 or 500 MPa), gels with very different spreadability were obtained, i.e., the lowest spreadability was obtained at 150 MPa and the highest at 500 MPa, of all the assessed samples induced with non-thermal methods. This might be the result of better packing of the molecules, with less spaces between the particles and thus limitation of their movements [24]. These observations are confirmed with the microstructures of analyzed hydrogels. Such statistically significant changes in spreadability were not observed in the case of HPH-induced gels in the I and II cycles.

### 3.3. The Influence of Induction Methods on Microrheology of Obtained Inulin Hydrogels

**Figure 6:**
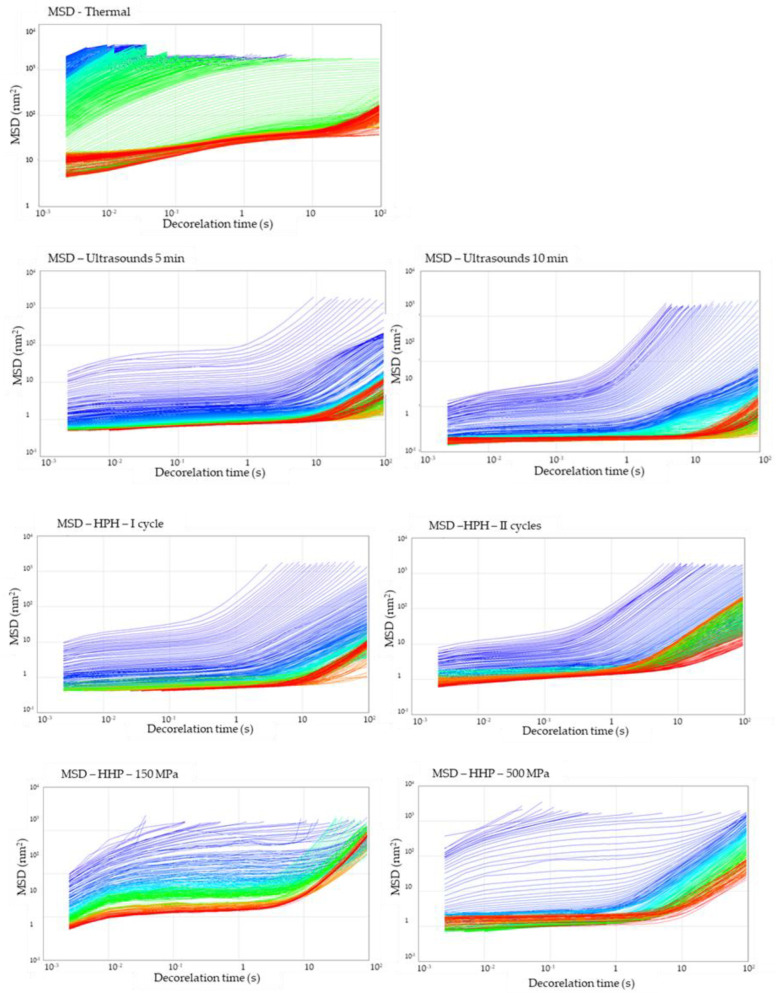
Effect of induction method on Mean Square Displacement (MSD) curves during gelation of aqueous solutions of inulin induced with the tested methods. The colors of the lines indicate the time elapsed from the start of the measurement (blue) to its completion (red).

The microrheological properties of the hydrogel samples under static conditions were measured using Rheolaser. Based on the analysis of the average trajectory by numerous particles, the Mean Square Displacement (MSD) was investigated as a function of time (Figure 6). All tested samples were not a purely viscous product (Newtonian product), particles in the samples could not move freely, and MSD curves were not a linear function of decorrelation time. As a result of the interaction of particles with each other, polymeric structures were created, preventing the particles from moving, and these interactions gave the elasticity to the product. It was observed that the gel structure in samples obtained with methods other than thermal was already partially formed during the process. Meanwhile, the profiles of the MSD curves for the gel obtained with the thermal method indicate that with time, the movement of the particles was slowed down (observation based on the course of the curves—initial—blue and then green), and after time, their complete immobilization in the gel network (red MSD curves reached a plateau) was detected. The reason why the gelation point was visible for the thermal induction method is that the gel structure is formed during the chilling of the solutions, whereas in other induction methods, the structure begins to form while the samples are still under the induction conditions [38]. The non-thermal gel samples underwent changes in the direction of increasing the elasticity of the samples; however, the strong three-dimensional structure was also obtained. What is more, for the samples induced with HPH (I and II cycle) and HHP (150 MPa) in the last stage, it is also observed that the MSD curves became linear again with decorrelation time, which might be a result of the partial particle release from the gel network.

Thermally induced hydrogels were characterized by the highest value of MSD (of the order > 10^3^ nm^2^) at the beginning of the analysis, and slightly lower values were obtained for gels induced by HHP (of the order < 10^2^ nm^2^). The use of ultrasound induction or high-pressure homogenization reduced the initial MSD value, and hence the initial mobility of molecules in hydrogels. The longest cross-linking time of molecules in the polymer matrix was found in the thermally induced hydrogel. The use of modern induction methods significantly reduced the time during which the movement of molecules was slowed down by the formation of intermolecular interactions.

**Figure 7:**
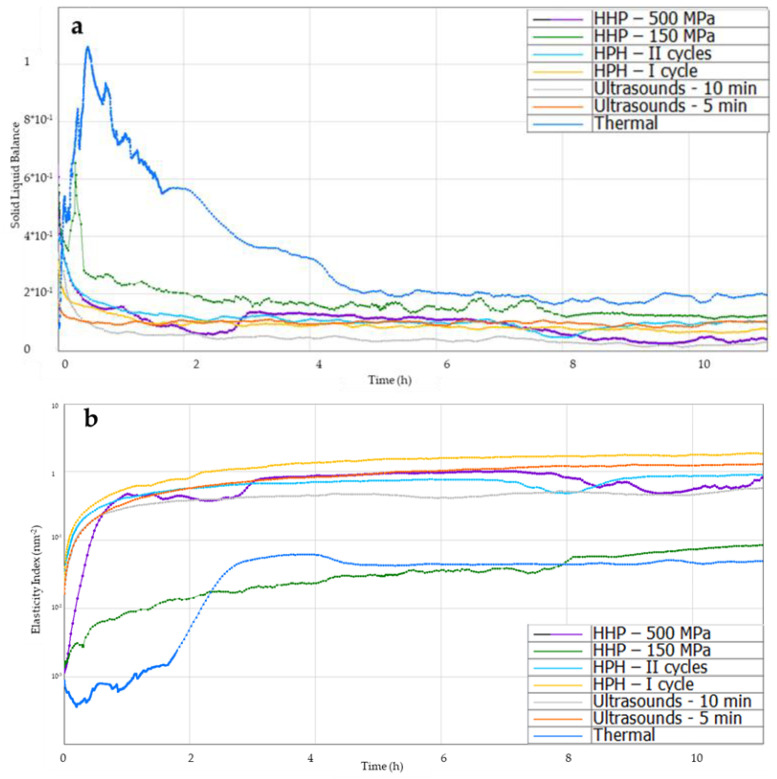

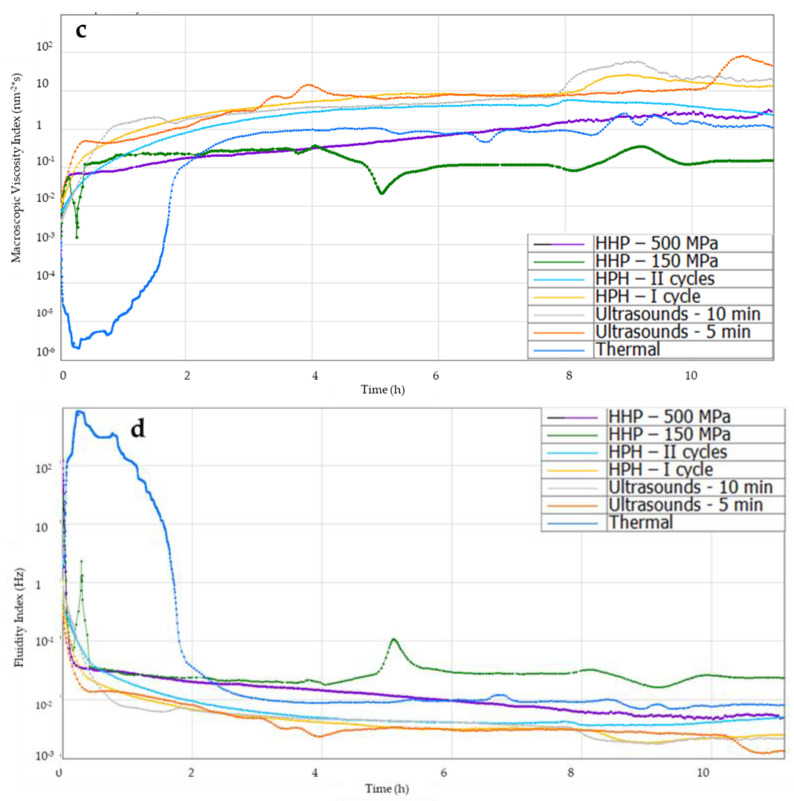
Effect of induction method on (**a**) solid–liquid balance (SLB), (**b**) Elasticity Index (EI), (**c**) Macroscopic Viscosity Index (MVI), (**d**) Fluidity Index curves during gelation of aqueous solutions of inulin induced with the tested methods.

Solid–liquid balance (SLB) allows for determining when the solid phase begins to dominate in the solution and the solution begins to gel, forming a hydrogel structure (Figure 7a). When this parameter is below 0.5, the tested sample acquires the characteristics of a solid body, while in the range from 0.5 to 1, it behaves like a liquid. Thermally induced hydrogels at the beginning showed the properties of a liquid, the graph shows a clear peak, and a longer time was needed (about 2 h) for the solid phase to dominate in the structure, while induction with ultrasound and homogenization allowed for obtaining this parameter below 0.5 at the beginning of the analysis, which proves the dominance of the solid phase immediately after induction of gelation of inulin solutions. Gels induced by high hydrostatic pressures, similarly to thermal gels, at the beginning of the analysis showed the properties of a liquid, but quite quickly (after about 20 min), the SLB value began to decrease, and no clear peak was noted for 500 MPa, which proves a shorter gel time compared to thermally induced gels.

Another investigated microrheological property of the gels was the Elasticity Index (EI) (Figure 7b). The Elasticity Index (EI) is proportional to the elastic modulus G′ at the plateau and gives an indication of the evolution of the elasticity of the product as a function of time [39]. The higher the EI, the higher the elasticity of a gel [40]. All novel induction methods, except HHP at the level of 150 MPa, allowed for obtaining higher EI values of hydrogels compared to those produced with the traditional (thermal) method. When examining the effect of the parameters of induction in individual methods, it was found that in the case of the use of HPH and ultrasound, changing the parameters (increasing process parameters) resulted in a slight decrease in the elasticity of the gels. In the case of HHP-induced gels, changing the process parameters, i.e., increasing the pressure from 150 to 500 MPa, resulted in obtaining gels with higher elasticity. At the same time, it was found that the use of the high-pressure gelation induction technique, depending on the parameters used, allowed for obtaining gels with the greatest diversity in terms of elasticity.

The Macroscopic Viscosity Index (MVI) allows for obtaining macroscopic viscosity curves (Figure 7c). It was found that up to the second hour after induction, the MVI of gels induced with non-thermal methods was higher than with the thermal method, which resulted from the subsequent formation of the gel structure. Analyzing the MVI curves 12 h after induction, it was found that the use of the ultrasonic method and HPH (first cycle) allowed for obtaining gels with a slightly higher MVI than in the case of traditional thermal induction. The lowest MVI was obtained for gels induced using HHP induction at 150 MPa. It was also found that increasing the pressure during HHP induction to 500 MPa resulted in obtaining inulin gels with higher viscosity, similar to thermally induced gels.

The influence of new induction methods on the elasticity and viscosity of hydrogels was also found by other authors. Bashash et al. [41], examining the induction of hydrogels with ultrasound, showed an increase in elasticity in comparison with thermal induction. In turn, Yu et al. [42], examining the effect of the ultrasound induction time, showed a clear increase in elasticity after doubling the time of exposure to ultrasound and a clear decrease in the elasticity of the hydrogels after increasing the induction time four times. Gelation induction with high-pressure homogenization was used by Shkolnikov Lozoberet et al. [21] and, similarly to our research, the authors showed that the use of HPH in the formation of hydrogels increased the elasticity of the samples. On the other hand, Opazo-Navarrete et al. [43], examining the rheological properties of aloe-based gels induced by high hydrostatic pressures, showed a decrease in elasticity at all tested pressures (300 MPa, 400 MPa, and 500 MPa) compared to the control test, in which the gel structure was created with a thermal method.

Analyzing the Fluidity Index (FI), it was found that within 2 h of induction, there were differences in FI between gels induced with non-thermal methods and the traditional (thermal) method (Figure 7d). Thermally induced gels were more fluid than gels induced using non-thermal methods due to the subsequent formation of a gel structure. Analyzing the gels 12 h after their induction, it was found that the use of new induction methods, such as ultrasound or high-pressure homogenization, reduced the value of the FI parameter. Gelation initiation times with ultrasound had no effect on this value, but the effect of the amount of cycles in high-pressure homogenization on FI was demonstrated. The use of two HPH cycles resulted in gels with a slightly higher FI than when HPH was used once. Such an influence of the applied induction process parameters on FI was also found in the case of induction using the HHP method. Induction with lower pressure (150 MPa) resulted in a higher FI value of hydrogels compared to gels in which the gelation process was caused by the application of pressure at the level of 500 MPa. Data available in the literature on the influence of different induction methods on the Fluidity Index confirm that the type of induction has an impact on this parameter. Similar dependencies were found, for example, by Bashash et al. [41] when examining the influence of ultrasound on the formation of protein hydrogels, or by Shkolnikov Lozoberet et al. [21] during research on the influence of HPH on the induction of spirulina-protein-based hydrogels and Opazo-Navarrete et al. [43] studying the rheological properties of aloe vera gels produced using high hydrostatic pressures.

### 3.4. The Influence of Induction Methods on Color of Inulin Hydrogels

On the basis of obtained results, it was found that all non-thermally induced hydrogels were characterized by the darker color, compared to the thermal method (lower L* value parameter) (Table 1). Generally, the use of different modern induction methods and various parameters of these processes did not significantly influence the value of the L* component. When analyzing the influence of induction parameters on the brightness of the gels, differences were found only in the case of the ultrasonic method. Gels produced with ultrasonication used for 5 min were significantly brighter compared to the samples obtained with a longer time of exposure to ultrasound. The lightness reduction by increasing ultrasonic treatment parameters was also found by Carrillo-Lopez et al. [44].

All tested hydrogels were characterized by negative values of the a* component (Table 1). It was found that all gels induced with non-thermal methods were characterized by significantly lower values of the color parameter a* than gels induced with the thermal method. Among the compared hydrogels, the gel induced with HPH II cycles had the lowest values of color parameter a*, and the highest was HHP at 500 MPa. Moreover, changing the induction parameters using non-thermal methods did not have a significant impact on the values of this color parameter.

All tested hydrogels were characterized by positive values of the b* component (Table 1). It was found that all gels induced with non-thermal methods were characterized by significantly lower values of the color parameter b* in comparison with gels induced with the thermal method. The use of various parameters in induction with modern methods resulted in obtaining hydrogels with significantly different values of this parameter. During ultrasonic treatment, the extension of the induction time significantly decreased the value of the b* component. In the case of high-pressure homogenization, extending the time of exposure of inulin solutions to HPH, i.e., the use of two cycles in the homogenizer, resulted in gels with a significantly lower b* component. Differences in the value of the b* component were also observed in hydrogels induced with different HHP parameters. The samples subjected to a pressure of 500 MPa were characterized by lower values of the b* component compared to the gels induced with a pressure of 150 MPa.

In order to comprehensively determine the impact of the induction method on the color of inulin gels, the parameter of the total color difference was also determined. Analyzing the obtained results, it was found that the color of all tested samples induced with modern induction methods was different from the gels produced thermally to such an extent that unexperienced observers notice the difference in colors (2 < Δ***E*** < 3.5) as, in a case of HHP 500 MPa, HPH I cycle, or 5 min Ultrasounds or even in a case of HHP 150 MPa, HPH II cycle, or 10 min Ultrasounds, a clear difference in colors is noticed (3.5 < Δ***E*** < 5) (Table 2).

The highest Δ*E* coefficient, and thus the greatest difference in the color of the gels, was found between the control sample and the gels induced with ultrasound for 10 min, while the smallest difference, but still visible to the observer, was demonstrated for samples induced with the pressure of 500 MPa. The influence of the induction parameters on the color difference of the obtained samples was also found for ultrasound induction and for the HHP induction; however, these differences will be noticed only by an experienced observer (1 < Δ*E* < 2). As for the induction method with high-pressure homogenization, no difference in color was found between the samples obtained in one or two cycles.

## 4. Conclusions

Based on the obtained results, it can be concluded that the analyzed non-thermal methods of inulin gel induction, i.e., ultrasound, pressure homogenization, and high-pressure technique, may be a good alternative to the traditional thermal induction method, especially for food that does not require heating. Additionally, the use of these non-thermal induction techniques offers many advantages over the thermal method. When they are used, the gel structure is created faster, and the gels are characterized by greater stability, lower solid–liquid balance, approach toward the solid phase, and, with the exception of HHP 150 induced gels, increased elasticity and viscosity index, as well as lower flow index. Gels produced using the tested non-thermal methods also have a different, more delicate and spreadable structure than thermally induced gels. Among the compared non-thermal methods of inulin gel induction, the high-pressure method has the greatest potential to modify the properties of the created hydrogels. Thanks to its application, it was possible to obtain gels with very different properties, both delicate (i.e., soft and spreadable), using HHP at 150 MPa, and hard, using HHP at 500 MPa, with properties most similar to gels induced with the thermal method. This may allow the properties of hydrogels to be matched to the characteristics of the food matrix being created.

## Figures and Tables

**Table 1:** The effect of induction method on color parameters of inulin hydrogels.

Induction Method	L*	a*	b*
Thermal	91.46 ^a^ ± 0.49	−5.56 ^a^ ± 0.06	5.96 ^a^ ± 0.11
Ultrasounds—5 min	88.72 ^b^ ± 0.38	−5.78 ^bc^ ± 0.03	4.27 ^b^ ± 0.25
Ultrasounds—10 min	87.09 ^c^ ± 0.46	−5.75 ^bc^ ± 0.07	3.80 ^cd^ ± 0.22
HPH—I cycle	89.26 ^b^ ± 0.35	−5.86 ^cd^ ± 0.03	3.82 ^cd^ ± 0.03
HPH—II cycles	88.65 ^b^ ± 0.09	−5.95 ^d^ ± 0.06	3.28 ^e^ ± 0.13
HHP—150 MPa	88.23 ^bc^ ± 0.01	−5.73 ^bc^ ± 0.04	4.17 ^bc^ ± 0.07
HHP—500 MPa	89.54 ^b^ ± 1.03	−5.72 ^b^ ± 0.02	3.75 ^d^ ± 0.09

The average (*n* = 3) values in columns with different letter symbols differ significantly (*p* < 0.05).

**Table 2:** Parameter of the total color difference between inulin hydrogels obtained using different induction methods ^#^.

Induction Method	HHP 500 MPa	HHP 150 MPa	HPH II cycles	HPH I cycle	Ultrasounds 10 min	Ultrasounds 5 min
Thermal	3.07	3.70	3.92	3.08	4.90	3.27
Ultrasounds 5 min	1.05	0.55	1.05	0.75	1.71	
Ultrasounds 10 min	2.46	1.22	1.67	2.18		
HPH I cycle	0.93	1.10	0.87			
HPH II cycles	1.11	1.01				
HHP 150 MPa	1.40					

# The color difference between the samples can be estimated based on the Δ*E* values [40]: if 0 < Δ*E* < 1, the color is determined as not noticeable for the observer; 1 < Δ*E* < 2, only experienced observers can notice the difference in colors; 2 < Δ*E* < 3.5, unexperienced observers also notice the difference in colors; 3.5 < Δ*E* < 5, clear color difference in colors is noticed; 5 < Δ*E*, observer notices two different colors.

## Data Availability

The data used to support the findings of this study can be made available by the corresponding author upon request.
